# Acoustoplasmonic Metasurfaces Based on Polymer-Grafted Nanoparticles

**DOI:** 10.1021/acs.nanolett.5c03009

**Published:** 2025-08-04

**Authors:** Thomas Vasileiadis, Anuj K. Dhiman, Adnane Noual, Nicholas Sbalbi, Matthew Ye, Robert J. Macfarlane, Bartlomiej Graczykowski, George Fytas

**Affiliations:** † Faculty of Physics and Astronomy, 49562 Adam Mickiewicz University, Uniwersytetu Poznanskiego 2, Poznan 61-614, Poland; ‡ Max Planck Institute for Polymer Research, Ackermannweg 10, 55128 Mainz, Germany; § LPMR, Department of Physics, Faculty of Sciences, Mohammed First University, Oujda 60000, Morocco; ∥ Department of Materials Science and Engineering, 2167 Massachusetts Institute of Technology, Cambridge, Massachusetts 02139, United States; ⊥ Department of Chemical Engineering, Massachusetts Institute of Technology, Cambridge, Massachusetts 02139, United States; # Institute of Electronic Structure and Laser, FORTH, N. Plastira 100, Heraklion 70013, Greece

**Keywords:** Acoustoplasmonics, metasurfaces, polymer-grafted nanoparticles, self-assembly, optomechanics, Brillouin light scattering

## Abstract

Nanocomposites assembled from polymer-grafted plasmonic nanoparticles (PGNs) can combine strong light-matter interactions with soft-matter functionalities and a high degree of translational symmetry. This work explored the potential of gold nanoparticles (16 nm diameter) grafted with polystyrene chains (degree of polymerization, *N* ≈ 63) as building blocks for acoustoplasmonic metasurfaces. We have decorated inorganic surfaces—crystalline silicon and SiO_2_ glass—with PGN monolayers and explored their surface acoustic waves with micro-Brillouin Light Scattering (μ-BLS) at various photon energies. Aided by finite-element-method calculations of acoustic phonons, plasmons and optomechanics, we show that PGNs sustain coupled sphere modes with rattling, torsional, and quadrupolar features. The coupled sphere modes exhibit plasmon-enhanced BLS and form a wide acoustic band gap below the line of sound. In the long wavelength limit, the coupling to the substrate leads to the emergence of shear-horizontal and Sezawa waves, whose dispersion relationships yield the local scale elasticity of ultrathin PGN monolayers.

## Graphical abstract

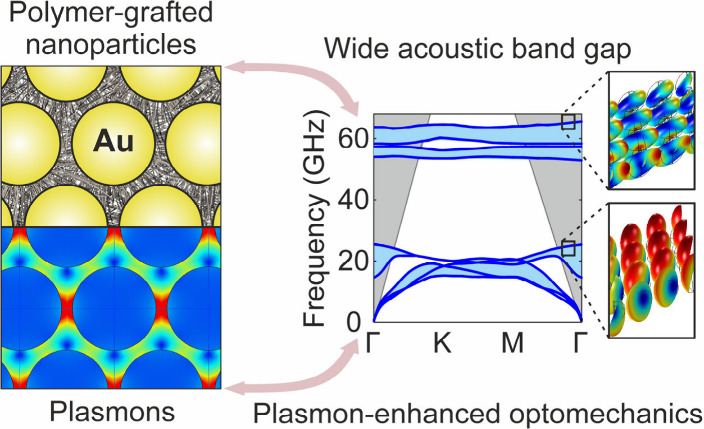

Polymer-grafted nanoparticles^1–4^ (PGNs) can synergistically merge the useful properties of inorganic nanomaterials with functionalities of soft matter, generating thermodynamically single-component hybrid structures that possess the functions of both organic and inorganic materials.^5–7^ On the one hand, the controlled architecture of the soft polymeric matrix surrounding the nanoparticles^8,9^ can offer mechanical flexibility,^10^ adhesive properties,^11^ combined functionalities from both polymeric and colloidal materials,^12^ and sensitivity to external stimuli like heat, exposure to solvents,^13^ or pressure.^11^ On the other hand, the selected nanoparticles can be made out of noble metals to sustain plasmonic resonances and enhanced light-matter interactions. Metal nanoparticle–polymer nanocomposites have been shown to exhibit plasmon-enhanced optomechanical interactions,^14–19^ while PGNs have served as prototypical plasmonic metamaterials.^20,21^ Moreover, the combination of enhanced light-absorption by plasmons and the malleability of the polymeric matrix make PGNs ideal materials for thermoplasmonic applications.^22,23^

Nanocomposites based on PGNs can be building blocks for macroscopic materials,^24^ and their mechanical properties can be engineered with focused ion beam milling.^25^ The high degree of translational symmetry offered by PGN nanocomposites^26^ renders them ideal candidates for phononic^27^ and plasmonic^20,28^ metamaterials. PGN nanocomposites can even serve as model systems for studying and tailoring the interactions between plasmonic and phononic degrees of freedom.^29,30^ PGN-based nanocomposites’ ability to generate macroscopically processable materials with both unique plasmon-derived optical properties and polymer-derived mechanical durability,^24^ makes them useful candidate materials for smart photodetectors,^31^ photonic devices for wavelength conversion^32^ or light-trapping,^33^ and neuromorphic visual systems.^34^

Here, we explore the potential of PGNs as building blocks for acoustoplasmonic metasurfaces,^35–38^ i.e., nanostructured surfaces that combine artificial band-gaps for surface acoustic waves with plasmon-enhanced optomechanical (OM) coupling of specific acoustic modes. To elucidate the phononic properties of ultrathin PGN nanocomposites, we decorated two inorganic surfaces, i.e., silicon (Si) and glassy silicon dioxide (SiO_2_), with monolayers of gold (Au) nanoparticles grafted with 6.5 kDa thymine-terminated polystyrene (PS) chains. The diameter of the Au nanoparticles is *D* = 16 nm with a standard deviation of 1.26 nm. The frequencies and dispersion relationships of the associated surface acoustic waves (SAWs) were examined with micro-Brillouin Light Scattering (μ-BLS) at various photon energies. The obtained results were interpreted with finite-element-method calculations of acoustic phonons, plasmons, and optomechanics.

The PGNs formed crystalline monolayers with uniform morphology over extended areas (on the order of 10^2^ μm^2^). The films were prepared via evaporation-driven assembly, in which 10 μL of a 90 nM nanoparticle solution in toluene was pipetted into the surface of a diethylene glycol (DEG) layer in a 1 cm diameter glass vial. Upon toluene evaporation, a nanoparticle monolayer formed on the surface of the DEG,^39^ which could be lifted off onto a desired substrate (Figure 1a). Remaining DEG was subsequently removed via vacuum drying. Further synthetic details are included in Supporting Information (SI) Section 1.

**Figure 1:**
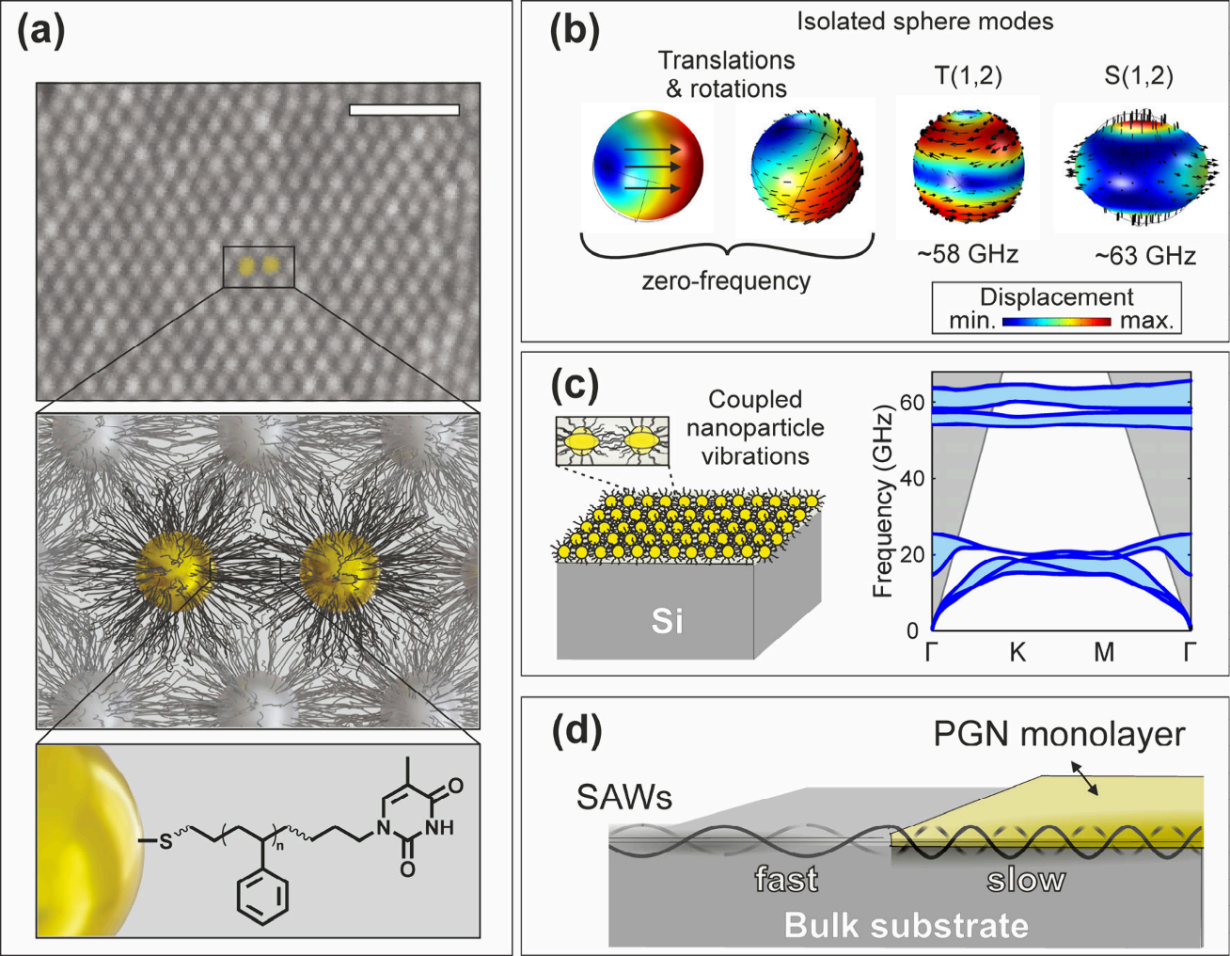
Phononic colloidal crystals formed by polymer-grafted nanoparticles (PGNs). (a) SEM imaging reveals the formation of hexagonally packed nanoparticle monolayers, with each Au particle grafted with thymine-terminated polystyrene. Scale bar: 100 nm. (b) The spherical Lamb modes of the isolated Au nanoparticle (*D* = 16 nm) in the absence of PS chains. The colormap shows the magnitude of the displacement and the black arrows indicate the direction of the displacement field. (c) In crystalline assemblies of PGNs the Lamb modes of the isolated nanoparticles form a band-structure (blue regions) along different directions of the reciprocal lattice (denoted as Γ, Κ, Μ for a hexagonal lattice). In PGN monolayers, nanoparticle translations and rotations become dispersive and acquire a nonzero frequency, while higher order modes form nearly flat bands. The gray areas show the line of sound (for Si), above which the phononic states are always including pseudosurface acoustic waves that leak to the bulk. (d) Due to the low transverse speed of sound in Au and PS (*c*_*t*_ ≅ 1200 m/s) the Rayleigh, longitudinal, and transverse surface acoustic waves of a stiffer substrate (like Si or SiO_2_) can transform into slower shear-horizontal and Sezawa waves.

In the absence of PS chains, an isolated Au nanoparticle with 16 nm diameter has torsional and spheroidal Lamb modes, such as the quadrupolar (1,2) mode (*n* = 1, l=2 ) at ∼63 GHz that is BLS active, or the torsional (1,2) mode at ∼58 GHz that is BLS inactive^40^ (Figure 1b). Moreover, the isolated nanosphere has zero-frequency translational and rotational motions that can acquire energy and BLS activity through mechanical and plasmonic coupling to adjacent nanospheres.^14,15,17,18^ For example, the frequency and BLS activity of the (1,1) Lamb mode can become different than zero when two nanoparticles are brought into contact.^41^ This coupled vibrational mode of nanoparticle dimers is often described as a “rattling” mode, and its BLS activity can be enhanced with plasmonic resonances.^14–19^ Additionally, the isolated nanoparticle has zero-frequency modes of rotational character, previously described as librations^17^ that may also become detectable with BLS in the presence of mechanical coupling and plasmonic resonances.^15^

While optomechanical coupling has been measured in the systems noted above, the terminologies mentioned so far are specifically used to describe nanoparticle dimers or disordered arrays of particles. For the PGN samples examined here (which contain long-range translational symmetry), the Lamb modes of the isolated nanospheres transform into a phononic band structure (Figure 1c). In this case, the mechanical coupling is mediated through the PS chains, and the Au surfaces are neither in direct physical contact with one another nor with the substrate.^42^ In the hexagonally packed crystalline monolayers, each particle is coupled to at most six nearest neighbors. Interestingly, both Au and PS have much lower transverse speed of sound (*c*_*t*_ ≅ 1200 m/s for both) than substrate materials like Si (*c*_*t*_ = 5840 m/s) and glassy/amorphous SiO_2_ (*c*_*t*_ = 3400 m/s). The deposition of a soft thin-film on top of stiffer substrates can lead to acoustic confinement and emergence of Sezawa modes (Figure 1d). As the phonon wavevector increases, the phase velocity of Sezawa waves decreases relatively to the Rayleigh SAWs of the bare substrate. In addition to Sezawa waves, the thin film can sustain shear-horizontal surface waves, which are also slower than the Rayleigh SAWs of the bare substrate.

To reveal plasmonic effects, the BLS spectra were recorded with two different wavelengths of light: λ = 532 nm and λ = 660 nm, which are respectively off- and on-resonance with the plasmons of the crystalline PGN monolayer. The experimental extinction spectra for the PGN monomers dispersed in toluene (green line, Figure 2a top) exhibited a narrow peak at ∼527 nm. For the crystalline PGN monolayer on glass, the peaks became significantly broader and red-shifted to ∼610 nm (solid red line, Figure 2a top), and hence the plasmons are nearly resonant with the 660 nm laser light (vertical red dashed line in Figure 2a). The PGN monolayers in the probed area of the sample (spot size ∼2 μm) are polycrystalline (Supporting Information S2). The experimental extinction spectra were accurately reproduced using finite-element-method (FEM) calculations (Figure 2a, bottom). Compared to the monomer, the monolayer has a broader and red-shifted extinction spectrum due to plasmonic coupling between neighboring nanoparticles, visualized in Figure 2b for 532 nm (top) and 660 nm (bottom). The maximum electric field norm, |*E⃗*|, between nanoparticles is doubled for 660 nm compared to 532 nm.

**Figure 2:**
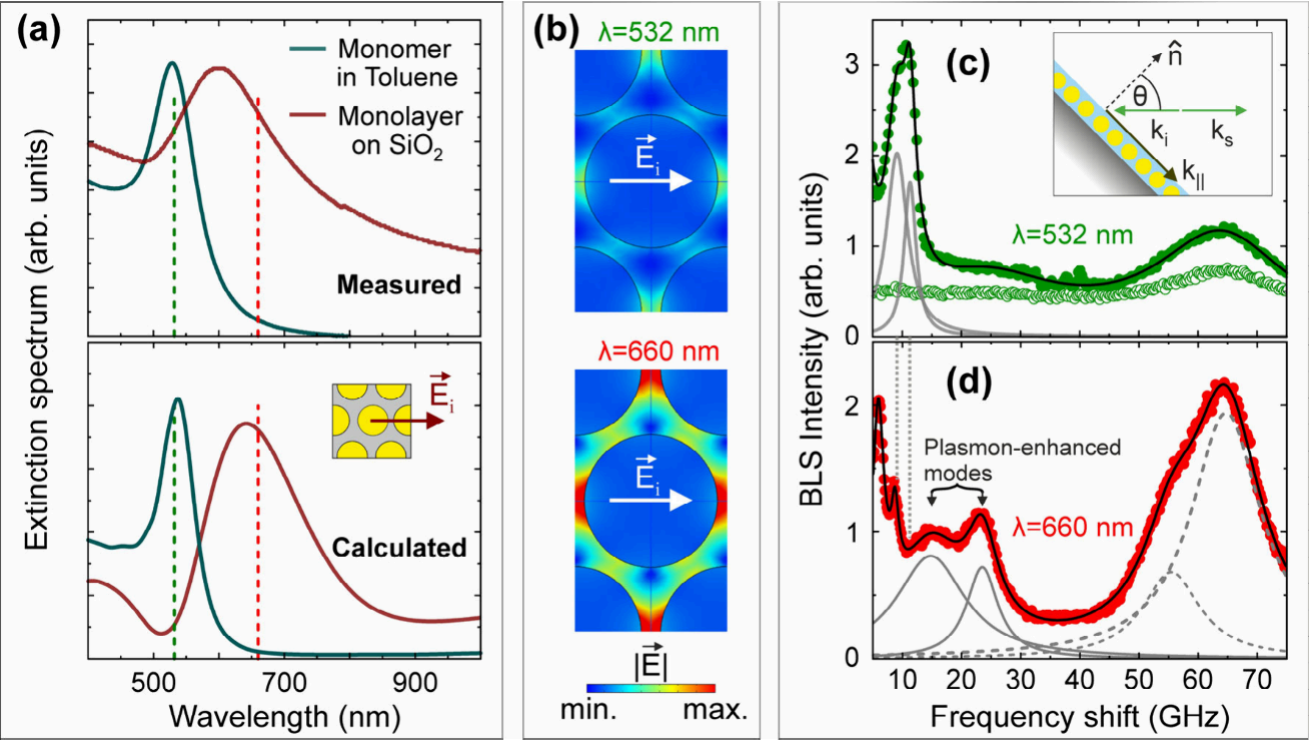
Brillouin light scattering (BLS) off- and on-resonance with the plasmons. (a) Measured (upper panel) and calculated (lower panel) extinction spectra of the monomers dispersed in toluene (green lines) and the monolayers deposited on glass (dark red lines). The FEM calculations of the extinction spectrum were performed for an incident electric field parallel to the [121] crystallographic direction of the FCC lattice, which is schematically shown in the inset of the lower panel. The vertical lines indicate the two wavelengths of light used for recording the BLS spectra: 532 nm (dashed green) and 660 nm (dashed red). (b) The electric field norm, |**E**|, for λ = 532 nm (top) or 660 nm (bottom). (c) Off-resonance: The experimental BLS spectrum on Si with HH polarization (full green circles) recorded at λ=532 nm wavelength of light, and its representation with a sum of Lorentzian peaks (solid black line). The intense signal at ∼10 GHz consists of two Lorentzian peaks (gray lines) at 9.1 and 11.3 GHz. These peaks do not appear in the experimental VH spectrum (open green circles). Inset: Scheme of the backscattering geometry showing the vector normal to the surface (**n̂**), the wavevectors of incident (***k*_i_**) and scattered (***k***_**s**_) light, and the phonon wavevector parallel to the surface (***k***_**||**_). (d) On-resonance: The experimental BLS spectrum on Si with HH polarization (full red circles) recorded at λ=660 nm, and its representation with a sum of Lorentzian peaks (solid black line). The low-frequency doublet in (c) is now shifted to lower frequencies (see vertical dotted lines). The rest of the spectrum consists of two plasmon-enhanced peaks at 14.8 and 23.5 GHz (gray solid lines), and a double peak at 55.5 and 64.7 GHz (gray dashed lines). The corresponding VH spectrum for Si is shown in Figure S3. For both (c) and (d) the angle of incidence was 45°, and the laser power was in the order of 3 mW.

The BLS spectra shown in Figures 2c (532 nm) and **2d** (660 nm) were recorded at backscattering geometry, which is schematically shown in the inset of Figure 2c. The probed phonon wavenumber parallel to the sample surface (*k*_||_) is given by *k*_||_ = 4π sinθ/λ, where θ is the angle formed by the vector normal to the surface (**n̂**) and the wavevectors of incident (***k*_i_**) and scattered (***k*_s_**) light. In systems with discrete translational symmetry like phononic crystals, the probed phonon wavevector can also be ***k***_||_ ± **G**, where **G** is a reciprocal lattice vector. In the measurements shown here (θ = 45°), *k*_||_ = 16.7 μm^–1^ for λ = 532 nm, and *k*_||_ = 13.5 μm^–1^ for λ = 660 nm. Since the BLS signal is generally low for a single monolayer of nanoparticles (*D* = 16 nm, effective thickness ∼20 nm) the measurements were performed with a wide collection angle at the expense of momentum resolution. Thus, the collected wavevectors span the interval 8.6–22.0 μm^–1^ for λ = 532 nm, and 7.0–17.7 μm^–1^ for λ = 660 nm.

The polarization of incident and scattered beams was horizontal-horizontal (HH) relative to the scattering plane (also termed p-p or TM-TM) so that surface acoustic waves can be detected. The BLS spectra (circles) in Figures 2c and 2d have been represented by a sum of Lorentzian peak profiles (black solid lines). To identify vibrational modes of nanoparticles that depolarize light, we have also performed measurements in VH (or s-p) polarization – see Figure 2c for 532 nm and Figure S3 for 660 nm light. Furthermore, in order to avoid irreversible laser-induced changes, all measurements were limited to maximum 3 mW laser power. The PGN monolayers on Si were very robust to laser irradiation and we did not find evidence for laser-induced heating, proving that the bulk Si wafer acts as an efficient heat sink. For PGN monolayers on SiO_2_ glass, which has significantly lower heat conductivity than crystalline Si, the laser power was reduced to ∼2.5 mW (Figure S4).

The off-resonance BLS spectrum with HH polarization (solid green symbols Figure 2c) consists of two intense and narrow peaks at 9.1 and 11.3 GHz (gray lines) as well as a broad high-frequency peak at 64 GHz. The latter is close to the quadrupolar (1,2) spheroidal mode of the single Au nanoparticle.^43^ The calculated frequency of the (1,2) mode for bare Au (*D* = 16 nm) nanoparticle in air is 63.5 GHz assuming isotropic elasticity (*c*_t_ = 1200 m/s, see also Figure S2). Note that anisotropic Au elasticity^44^ would lead to a much lower frequency of the (1,2) mode (46.6 GHz in Figure S2). The addition of a 2.35 nm PS layer on the Au surface redshifts the (1,2) mode to 62.4 GHz according to FEM calculations using isotropic elasticity of Au, and Young modulus (*E*_*PS*_ = 4 GPa), density (ρ = 1040 kg/m^3^) and Poisson’s ratio (*ν*_*PS*_ ≅ 0.34) of bulk PS^45^ (*M*_w_ = 61800, *M*_w_/*M*_n_ = 1.03). The FEM calculations, assuming anisotropic elasticity of Au and 2.35 nm PS on its surface, predict *f*_1,2_ = 46 GHz (SI Section 2). Therefore, the investigated particles here are certainly polycrystalline, have a spherical shape,^46^ and the Au elasticity is isotropic in contrast to previous studies on Au nanorods.^15^

For the off-resonance depolarized VH spectra (open green circles, Figure 2c), the low-frequency double peak vanished, but the high-frequency peak was preserved albeit with lower signal. Based on these observations, we attribute the peaks at 9.1 and 11.3 GHz to propagating surface acoustic waves (SAWs), which are BLS active only for HH polarization, and the peak at 64 GHz to quadrupolar-like sphere modes. Noticeably, the BLS spectrum with HH polarization may also contain a weak and broad peak at ∼24 GHz (Figure 2c), whose intensity is almost comparable to the measurement’s noise level.

To get a more detailed view of the BLS-active modes of the sample, the BLS spectrum was recorded at HH polarization (full red circles) using light that was on-resonance with the plasmons (λ=660 nm) (Figure 2d); its representation is also presented as a sum of Lorentzian peaks (solid black line). Due to the longer wavelength of light (lower *k*_||_), the SAWs are now shifted to lower frequencies, i.e., at 6 and 8.8 GHz. Moreover, the high-frequency peak now appears as a double Lorentzian with components at 55.5 and 64.7 GHz (dashed gray lines). Another striking feature of the on-resonance BLS spectra is the emergence of two extra peaks at 14.8 and 23.5 GHz (solid gray lines). The intensity of these two peaks is about 45% of the high-frequency spectrum. Although the absolute enhancement factor of the on-resonant spectra compared to the off-resonant cannot be quantified without considering the light dispersion in all optical elements of our setup, the emergence of new modes is a clear indication of plasmonic effects on optomechanics and BLS. Based on previous works^14,15,17,18^ and the wavelength-dependence of plasmonic fields shown in Figure 2b, the low frequencies 14.8 and 23.5 GHz can be straightforwardly assigned to mechanically coupled nanoparticle modes with plasmon-enhanced OM coupling. This assignment will also be supported by FEM calculations (*vide infra*).

Having established an understanding of the acoustic peaks and their interpretation, the dispersive SAW peaks with momentum-resolved BLS can also be examined. As shown in Figures 2c, 2d, the HH spectra can reveal both SAWs and vibrations of mechanically coupled nanoparticles. The depolarized BLS VH spectra—see for instance Figure S3—could be to identify nanoparticle vibrations. To study SAWs, the ideal polarization of incident and scattered light is HH. To record the HH spectra of SAWs, the collection angle was significantly narrowed for high-resolution measurements. Figure 3a shows BLS spectra recorded at 532 nm (upper panel, green symbols) and 660 nm (lower panel, red symbols) at a representative wavevector, *k*_||_ ≅ 13.3 μm^–1^, corresponding to an angle θ of 34° for 532 nm and 44° for 660 nm light. The experimental spectra were again reproduced with Lorentzian peak profiles (solid black lines). To obtain the dispersion relationship of SAWs, the extracted frequencies were plotted as a function of *k*_||_ (Figure 3b). The observed SAWs have lower frequencies (velocities) than the longitudinal bulk acoustic waves (LBAWs, gray dashed line), the transverse bulk acoustic waves (TBAWs, gray dashed-dot line), and the Rayleigh surface acoustic waves (RSAWs, gray dotted line). The fact that the detected SAWs have significantly lower frequencies than bulk substrate waves of the same wavevector clearly indicates their sensitivity to the elastic properties of the PGN monolayer atop.

**Figure 3:**
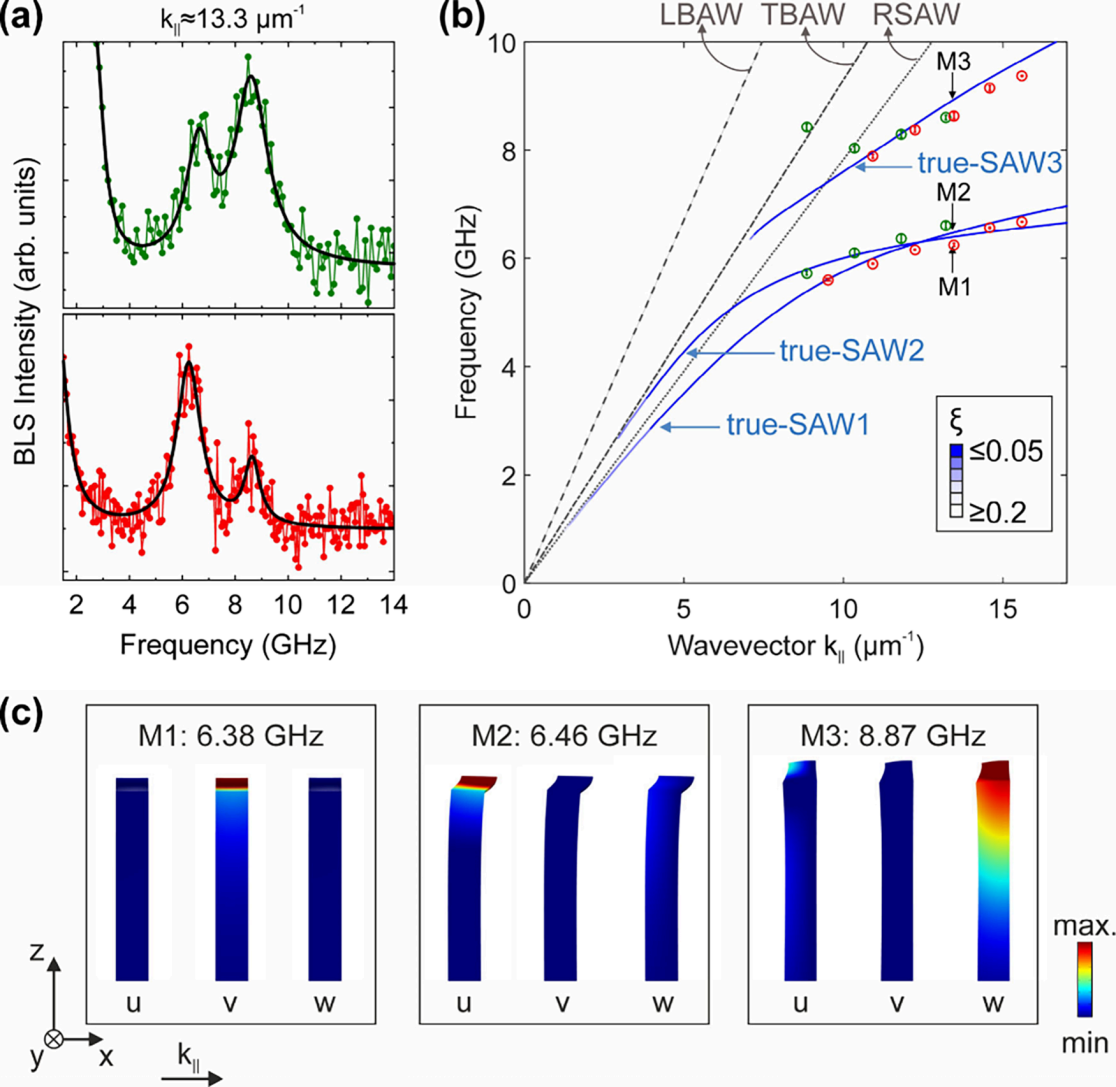
Surface acoustic waves of nanoparticle monolayers on silicon substrate. (a) BLS spectra measured with HH polarization at *k*_||_∼13.3 μm^–1^ with 532 nm (up) and 660 nm (down) wavelength of light. The corresponding angle θ is 34° for 532 nm and 44° for 660 nm light. The experimental spectra are represented with Lorentzian peak profiles (black solid lines). (b) Calculated dispersion relationships for Rayleigh surface acoustic waves, RSAW (gray dotted line) without Au-PS PGN nanoparticles, and true surface acoustic waves (true-SAW) with nanoparticles (blue curves). For comparison, the same graph shows the dispersion relationships for longitudinal bulk acoustic waves (LBAW) and transverse bulk acoustic waves (TBAW). The symbols denote the experimental data recorded with 532 nm (green circles) and 660 nm (red circles). The blue color of calculated acoustic modes in the presence of nanoparticles is weighted by a factor ξ (see inset and main text for more details) that filters out the pseudosurface acoustic waves. For true surface acoustic waves ξ < 0.2 by definition. The black arrows indicate the frequency of the first three true-SAWs at *k*_||_ = 13.3 μm^–1^ denoted as M1, M2, and M3. (c) The components of the displacement field (*u*, *v*, *w*) corresponding to the (*x*, *y*, *z*) directions in space for the M1, M2, and M3 true-SAWs. All modes have a significant in-plane motion (*u* or *v* component), with M1 and M2 representing almost exclusively in-plane motions.

In order to interpret the results of Figure 3b, FEM calculations were performed, in which the crystalline monolayer was treated as a homogeneous thin film. In these calculations, we needed to distinguish the true surface acoustic waves (true-SAWs) from the pseudosurface acoustic waves that are partially leaking to the bulk.^47^ This was achieved by calculating a parameter ξ accounting for localization of elastic energy close to the free surface.^47^ As ξ → 0, the acoustic mode becomes a true-SAW, while for ξ > 0.2 the acoustic mode becomes a pseudo-SAW. The calculated band structures in Figure 3b (blue shaded points) are weighted by this ξ factor. To reproduce the experimental results, we used an effective monolayer thickness (21.7 nm), density ρ=7016 kg/m^3^, Poisson ratio ν=0.37, and obtained a Young’s modulus *E* = 7.3 GPa. The exact same parameters of the effective thin film reproduced the dispersion of true-SAWs on SiO_2_ glass (SI Section 6)).

The derived elasticity of the PGN monolayer shows that the nanocomposite is approximately obeying the inverse Wood’s law of mixtures, which is a lower bound of *E* compared to the upper bound represented by a linear rule of mixtures. We notice that the reported *E* value (1.49 GPa) for a thick film of the same PGN measured with AFM indentation^25^ is even lower than the PS Young’s modulus, which cannot be explained by the inverse Wood’s law of mixtures. A possible explanation is that this low value represents the modulus normal to the film,^48^ while the value extracted from BLS is sensitive to mechanical deformations parallel to the surface. Indeed, the BLS measurements probe true-SAWs with a significant in-plane motion, as it is evident from the calculated displacement fields of Figure 3c.

The displacement fields depicted in Figure 3c were calculated for the same parallel wavevector (*k*_||_ = 13.3 μm^–1^) as the BLS spectra of Figure 3a. The true-SAWs at *k*_||_ = 13.3 μm^–1^ that contribute to the BLS signal are shear-horizontal waves at 6.38 GHz (M1), Sezawa waves at 6.46 GHz with primarily longitudinal motion (M2), and Sezawa waves at 8.87 GHz with primarily transverse motion (M3). The M1 and M2 waves correspond to predominantly in-plane motions, while the M3 has stronger out-of-plane motion (last panel of Figure 3c). Notably, this higher frequency mode contributes weaker to BLS signal at 660 nm compared to 532 nm (Figure 3a). An explanation for this trend is that the plasmon-enhanced BLS signal is more sensitive to in-plane motions that modify the interparticle distances causing stronger modulations of plasmonic near fields (Figure 2b). In the case of the opaque Si substrate irradiated with 532 nm photons, the substrate can also contribute to the BLS signal through the moving interface effect, which requires out-of-plane motion (w-component of displacement). In agreement with this explanation, the same mode is less intense for the transparent glass substrate, which would be expected to have a weak moving interface effect (Figure S5). Therefore, the Young’s modulus *E* value of the PGN monolayer extracted from BLS can differ from the prior values obtained with nanoindentation, because BLS probes low-frequency shear-horizontal and Sezawa waves with strong in-plane displacement.

We now return to the origin of the plasmon-enhanced PGN modes (Figure 2c,d and Figures S3 and S4). The frequencies off- and near-resonance, summarized in Table 1, can be used to identify the modes by comparison with FEM calculations. In prior works, these calculations were performed for mechanical and plasmonic coupling in nanoparticle dimers embedded in polymer matrices to explain the emergence of BLS peaks that are absent for bare nanoparticles.^14,15,17,18^ In Supporting Information S8, we present theoretical calculations of OM coupling in Au nanoparticle dimers embedded in a PS matrix (Figure S7a). The mechanical coupling leads to rattling and torsional modes in the frequency range of 10–25 GHz (Figure S7b) and multiple coupled quadrupolar modes between 60 to 70 GHz (Figure S7c). The plasmon-enhanced OM coupling gives BLS peaks at approximately 16 and 65 GHz with rattling and quadrupolar characters, respectively. Expectedly, the FEM calculations for the dimer cannot explain all four modes (Table 1), requiring that the FEM calculations be extended for the translationally symmetric PGN monolayers.

**Table 1:** Vibrational Frequencies of the Polymer-Grafted Nanoparticles for Different Wavelengths of Light and for Different Substrates, Shown Together with the Employed Polarization for BLS Measurements

		Plasmon-enhanced peaks (GHz)		High-frequency peaks (GHz)		
Substrate	Wavelength (nm)					Polarization
Silicon	532				64	HH
	660	14.8	23.5	55.5	64.7	HH
		14.3	22.4	56.4	64.4	VH
SiO_2_ glass	532				63.8	HH
	660	15	22.3	56.7	64.6	HH
		16.6	23.5	56	65.4	VH

The band structure, i.e., the eigenfrequencies as a function of *k*_||_, is calculated along the high-symmetry points (Γ-Κ-Μ-Γ) of the hexagonal lattice (Figure 4a). All modes below 50 GHz and under the line of sound acquire nonzero frequency due to interactions mediated by the polymer matrix. The high-frequency modes (>50 GHz), however, are reminiscent of the individual Au nanospheres. In this way, the PGN monolayer acts as a wide-band gap (∼30 GHz) two-dimensional phononic crystal. The calculated displacement fields (Figure 4b) show that the low-frequency (<30 GHz) branches are dispersive rattling and torsional modes, while the nearly flat bands at ∼58 and 65 GHz have torsional and quadrupolar features, respectively. Such flat bands offer the possibility of enhanced OM interactions through localization of elastic energy.^49^

**Figure 4:**
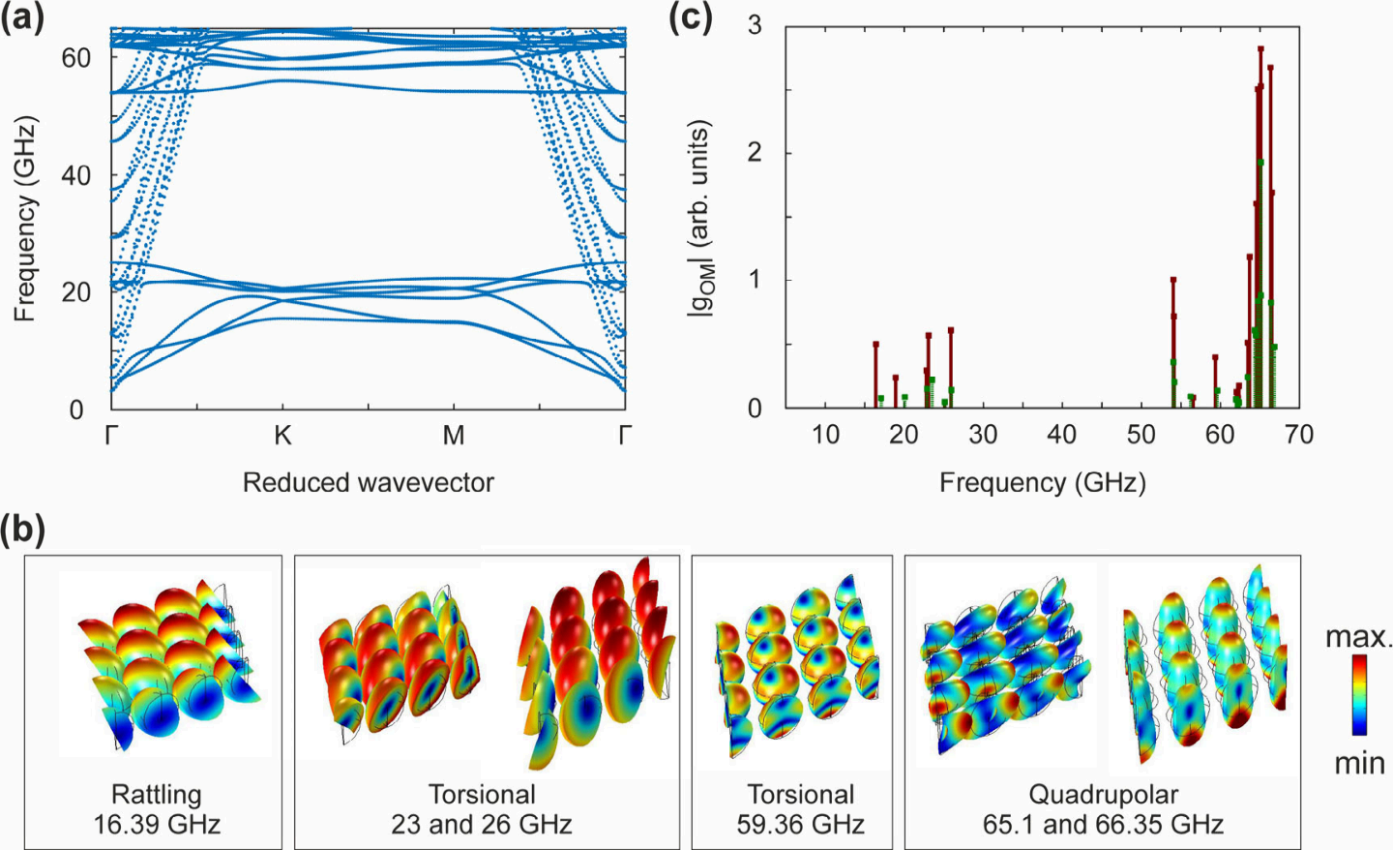
Band structure and optomechanical coupling of coupled nanoparticle modes on silicon. (a) The band structure of coupled PGN modes across the high-symmetry *k*-points of the hexagonal lattice. (b) The displacement fields of the BLS active modes and their patterns: rattling at 16.39 GHz, torsional at 23 and 26 GHz, higher-order torsional at 59.36 GHz, and quadrupolar at 65.1 and 66.35 GHz. The parallel wavevector is set to *k*_||_ = 13.5 μm^–1^ in the Γ-to-Μ direction. The colormaps indicate the mean displacement. (c) The optomechanical coupling strength, |*g*_OM_|, for 532 nm light (green stems) and 660 nm (red stems). For these calculations we used *k*_||_ = 16.7 μm^–1^ for λ = 532 nm, and *k*_||_ = 13.5 μm^–1^ for λ = 660 nm.

The OM coupling has two contributions: (i) the moving interface (MI) effect on the surface of the nanoparticles, and (ii) the photoelastic (PE) effect in the nanoparticle volume.^14,15,50–53^ Expectedly, the MI effect yields the strongest contribution for plasmonic nanostructures,^14,15^ where the surface-to-volume ratio is higher. The MI contribution is approximated by^15,54,55^

$$g_{MI}\propto \frac{\omega_{o}}{2}\iint u\cdot n\left(E_{s∥}\delta \varepsilon E_{s∥}-D_{s⊥}\delta \varepsilon^{-1}D_{s⊥}\right)\;dS$$

where the integration is performed over the Au surface. In this expression, **u** is the normalized displacement field, **E**_s∥_ is the tangential component of the scattered electric field, and **D**_s⊥_ is the normal component of displacement field. *δε* = *ε*_1_ - *ε*_2_ and *δε* ^–1^ = *ε*_1_^–1^ - *ε*_2_^–1^ are the differences in the permittivities of the nanoparticle (*ε*_1_) and surrounding (*ε*_2_) media. The calculated optomechanical coupling strength for every eigenmode is shown in Figure 4c. The OM coupling strength in Figure 4c was calculated for the same *k*_||_ as in Figures 2c, 2d (*k*_||_ = 16.7 μm^–1^ at λ = 532 nm, and *k*_||_ = 13.5 μm^–1^ at λ = 660 nm). Without adjustable parameters we obtain good agreement between the results of Figure 4c and the frequencies of Table 1. The nearly flat bands above 50 GHz have the strongest OM coupling.

Utilizing polymer-grafted Au nanoparticle monolayers (21.7 nm-thick) on two different substrates, we examined acoustoplasmonic behavior with momentum-resolved μ-BLS at two different wavelengths of light excitation. The dispersive properties of Sezawa waves provide a nonlocal and nondestructive probe of the monolayer elasticity predominantly parallel to the surface. The obtained value of Young’s modulus (7.3 GPa) is close to the lowest bound limit (Wood’s law) and considerably higher than the value reported for deformations normal to the film (∼1.5 GPa). In addition to Sezawa waves, dispersive sphere modes with rattling and torsional patterns are resolved at 15 to 24 GHz. These low-frequency branches are separated by a 30 GHz-wide band gap from localized sphere modes. The former is sensitive to mechanical coupling between nanoparticles mediated by polystyrene chains, whereas the later are much less sensitive to the matrix elasticity. The small frequency splitting and the near-flat dispersion of the coupled torsional and coupled quadrupolar modes indicate that acoustic hybridization mediated by grafted polymer chains is weak for high-frequency vibrations. In resonance conditions (λ=660 nm), BLS is plasmonically enhanced, since sphere modes modify the interparticle gaps causing strong modulations of plasmonic near fields. The plasmon resonances enable the BLS detection of torsional modes. Finally, the broad two-dimensional band gap as well as the existence of nearly flat bands enable several potential applications,^56^ e.g., vibrational isolation of supported nanodevices on surfaces, and localized resonators with plasmon-enhanced optomechanical coupling.

## Supplementary Material



## Notes

Information about the synthesis of polymer, Au nanoparticles, and nanoparticle monolayers, characterization with scanning electron microscopy, eigenmodes of single nanoparticles, depolarized BLS spectra of nanoparticles on Si, BLS spectra of nanoparticles on SiO_2_ glass, dispersion of SAWs for nanoparticle monolayers on SiO_2_ glass, BLS measurements of bare (1-dodecanethiol-capped) Au nanoparticles, FEM calculations of dimers of nanoparticles, additional information on the photoelastic contribution to optomechanical coupling, details on the FEM calculations of phononic band structures, and optomechanical calculations for nanoparticle monolayers on SiO_2_ glass (PDF)
