# Supplementary material for: Acoustoplasmonic Metasurfaces Based on Polymer-Grafted Nanoparticles
Source: Nano Lett. 2025 Aug 4;25(32):12351–9. doi: 10.1021/acs.nanolett.5c03009 (PMC12356073; doi:10.1021/acs.nanolett.5c03009)
Supplement: Supplementary file 1 [file nl5c03009_si_001.pdf]

Supporting Information:
Acoustoplasmonic metasurfaces based on polymer-grafted nanoparticles

Thomas Vasileiadis*[1,2†], Anuj K. Dhiman[1,2†], Adnane Noual[3], Nicholas Sbalbi[4,5], Matthew Ye[4], Robert J. Macfarlane[4], Bartlomiej Graczykowski[1,2], George Fytas*[1, 2, 6]

1. Faculty of Physics, Adam Mickiewicz University, Uniwersytetu Poznanskiego 2, Poznan, 61–614 Poland
2. Max Planck Institute for Polymer Research, Ackermannweg 10, 55128 Mainz, Germany
3. LPMR, Department of Physics, Faculty of Sciences, Mohammed First University, Oujda, 60000, Morocco
4. Department of Materials Science and Engineering, Massachusetts Institute of Technology, Cambridge, Massachusetts 02139, United States
5. Department of Chemical Engineering, Massachusetts Institute of Technology, Cambridge, Massachusetts 02139, United States
6. Institute of Electronic Structure and Laser, FORTH, N. Plastira 100, Heraklion, 70013 Greece

†Equal contributions

* thomas.vasileiadis@amu.edu.pl , fytas@mpip-mainz.mpg.de

S1: Synthesis of polymer-grafted Au nanoparticles.

S1.1 Polymer Synthesis

The polymer used in this work was 6.5 kDa polystyrene modified on one end with a thiol group to enable grafting to the Au nanoparticle surface and on the opposite end with a thymine group (Thy-PS-SH). This polymer was used because it was found to yield higher quality monolayers than polystyrene of similar molecular weight without end-modification. The polymer was synthesized according to previously reported procedures[1–3], involving atom-transfer radical polymerization (ATRP) from a thymine-based initiator and substitution of the bromine terminus with a thiol group, as described below.

The thymine-based ATRP initiator (1-(11-(2-bromo-2-methylpropionyloxy)undecyl)thymine) was synthesized in two steps. In the first step, 5.0 g of thymine (40 mmol), 1.0 g of 11-bromoundecan-1-ol (4 mmol), 1.1 g of potassium carbonate (8 mmol), and 200 mL of dimethyl sulfoxide were combined in a round bottom flask. The mixture was stirred and degassed with nitrogen for 2 min, then sealed with a septum and stirred for 48 hours. The mixture was then extracted in water and chloroform. The organic phase was dried with sodium sulfate, and the solvent was removed by rotary evaporation. The solid product was recrystallized in a mixture of 200 mL of ethyl acetate and a small amount of hexanes to afford 1-(11-hydroxyundecyl)thymine as a white solid. In the second step, 592 mg of 1-(11-hydroxyundecyl)thymine (2 mmol) was dissolved in 150 mL of dichloromethane with gentle heating, and 0.39 mL of triethylamine (2.8 mmol) was added to the solution. The mixture was

cooled to 0 °C, and a solution of 552 mg of α-bromoisobutyryl bromide (2.4 mmol) dissolved in 10 mL of dichloromethane was added dropwise under nitrogen. The reaction mixture was allowed to warm to room temperature and stirred for 14 hours. The mixture was then washed with sodium carbonate (2x) and brine (2x). The organic phase was dried with sodium sulfate, and the solvent was removed by rotary evaporation. The thymine initiator was purified by flash chromatography using an 8:3 mixture of dichloromethane and ethyl acetate.

ATRP was carried out by combining the thymine initiator (1.0 eq), styrene (200 eq), N,N,N',N'',N'''-pentamethyldiethylenetriamine (0.83 eq), and anhydrous anisole (0.3:1 v/v to styrene) in a dry Schlenk flask. The mixture was taken through 3 freeze-pump-thaw cycles and refilled with nitrogen. Copper(I) bromide was quickly added to the flask under a strong nitrogen flow, and the flask was immediately resealed and immersed in a 110 °C oil bath with stirring. The progress of the reaction was monitored by performing gel permeation chromatography (Agilent 1260 Infinity II) on aliquots drawn from the reaction mixture, and upon reaching the target molecular weight, the reaction was quenched by pouring the mixture into cold methanol, redissolving in dichloromethane, and precipitating in cold methanol again. The thymine-terminated polystyrene (Thy-PS) was dried under vacuum.

Finally, thiol end-modification of Thy-PS was carried out by combining Thy-PS (1 eq), 2-aminoethanethiol (15 eq), triethylamine (40 eq) and anhydrous DMF (~10-15 mL per 1 g of polymer) in a round bottom flask. The reaction mixture was degassed with nitrogen for 5 minutes and left stirring at room temperature for 60 hours. It was then poured into methanol, redissolved in dichloromethane, and precipitated in methanol again. The Thy-PS-SH product was dried under vacuum.

S1.2 Gold Nanoparticle Synthesis

Citrate-capped 16 nm Au nanoparticles were synthesized according to previously reported seeded growth protocols, in which ~10 nm Au nanoparticle seeds were first synthesized, then grown to 16 nm[4–6]. In brief, 485.3 mg of trisodium citrate dihydrate (1.65 mmol), 115.6 mg of citric acid monohydrate (0.55 mmol), and 800 mL of Milli-Q water were combined in a round bottom flask cleaned with aqua regia. The solution was heated in an oil bath while vigorously stirring; once boiling, 1 mL of a 14.6 mM solution of ethylenediaminetetraacetic acid tetrasodium salt dihydrate in Milli-Q water was added. Next, 3 mL of a 44.4 mM solution of hydrogen tetrachloroaurate(III) trihydrate in Milli-Q water was swiftly injected to the reaction mixture, causing a color change to red, indicating the formation of ~10 nm Au nanoparticle seeds.

When no further color change could be observed, the temperature was reduced to 90 °C and the reaction mixture was heated for an additional 30 mins to allow for thermal equilibration. Then, two additional injections of 44.4 mM hydrogen tetrachloroaurate(III) trihydrate were carried out with a 30 min equilibration period between each injection. The red color darkened after each injection, indicating the growth of the Au nanoparticles. The reaction mixture was then cooled to room temperature and stored as a stock solution at 4 °C without further purification.

S1.3. Gold Nanoparticle Functionalization with Thy-PS-SH

To functionalize Au nanoparticles with Thy-PS-SH, 7.7 mg of Thy-PS-SH was first dissolved in 10 mL of acetone. After dissolving, the polymer solution was added to 10 mL of the previously described Au nanoparticle solution (4.6 nM), and the mixture was shaken vigorously on a vortexer for 20 minutes. After shaking, the mixture, which contained grafted nanoparticle aggregates, was centrifuged briefly (3 min, 3000 rcf) and the supernatant was carefully removed. The resulting solids were then dissolved in a minimal amount of dimethylformamide (DMF) until they resuspended (~7 mL). The resulting dispersion was split into 1.5 mL Eppendorf tubes and then washed three times using 50 minute, 4600 rcf centrifugation cycles, between which the supernatant was replaced with 1) 1.2 mL DMF 2) 1.2 mL toluene and 3) 0.2 mL toluene. The dispersions were then recombined, and the final solution concentration was determined via UV-Vis spectroscopy.

S1.4. Preparation of Dodecanethiol (DDT) Grafted Au Nanoparticle Monolayers

Nanoparticles functionalized with significantly shorter dodecanethiol grafts were utilized as a control. Films were prepared using a technique adapted from Park et al. in which Au particles are simultaneously grafted and assembled at a liquid-liquid interface[7]. First, 800 μL of the previously described citrate-capped Au solution in water (4.6 nM) was added to a 1.5 cm perfluoroalkoxy alkane (PFA) well (Savillex), covering a piece of the desired substrate. Next, 400 μL of a 0.4 μM DDT solution in hexane was pipetted onto the surface of the nanoparticle solution, forming a stable upper layer. The well was then covered to prevent airflow disturbances and a syringe pump was used to add 240 μL of ethanol (EtOH) to the bottom of the well over the span of 4 min. The addition of EtOH caused the spontaneous migration of nanoparticles to the interface, at which they become grafted with DDT. Following EtOH addition, the syringe pump was reversed, slowly withdrawing the water layer over 20 min and lowering the film onto the substrate. The film was then dried overnight to remove any remaining solvent.

S2: Morphology of polycrystalline Au-PS monolayer.

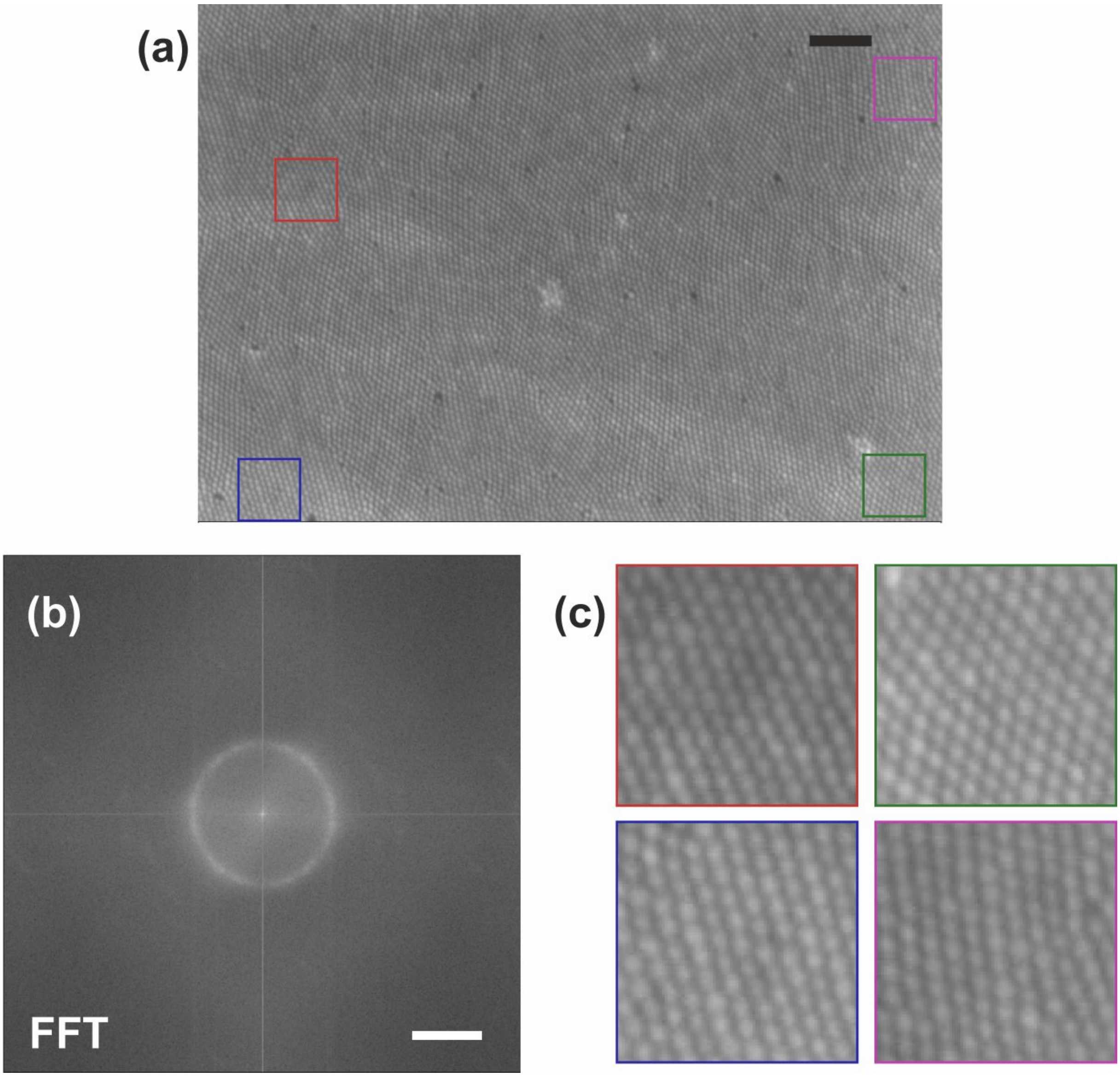

Figure S1: Electron microscopy images and Fourier transform of polycrystalline PGN monolayers. (a) Low-magnification scanning electron microscopy (SEM) image of PS grafted Au on Si substrate. The scale bar of the SEM image is 200 nm. **(b)** Fourier transform of the SEM image showing Debye rings due to polycrystallinity. The scale bar for the Fourier transform is 0.6 nm^{-1}. **(c)** Examples of crystalline domains with different orientations corresponding to the areas marked with squares in **(a)** (same color). The zoomed in images in **(c)** are 200 nm wide.

The low-magnification SEM image of **Figure S1a**. Evidently, the polymer-grafted nanoparticles (PGNs) form a closed homogeneous monolayer over extended areas. However, the Fourier transform of this image, shown in **Figure S1b**, reveals that the monolayer has a

polycrystalline structure. Thus, the diffraction spots transform into Debye rings due to the multiple orientations of the individual domains. Some examples of these domains are shown in the zoomed-in panels of **Figure S1c**. These images are 200 nm wide, and they correspond to the same-colour squares of **Figure 1a**. The size of the crystalline domains is in the order of ~200-300 nm, while the distance between every particle and its nearest neighbours is 20.7 nm, i.e., the 16 nm diameter of Au nanoparticles plus a 2.35 nm-thick surface layer of PS chains. Based on the Fourier transform, we conclude that the ring observed in **Fig. S1b** corresponds to (220) reflections of the hexagonal lattice with real-space distance of ~10.36 nm, and that the crystalline domains are randomly oriented.

S3: Lamb modes of bare and PS-grafted Au nanoparticles in air.

The quadrupolar frequency of the bare Au at 63.6 GHz is well captured by the $f(1,2)=0.85c_t/D_{Au}=63.8$ GHz where $c_t=1200$m/s is the transverse sound velocity of Au[8] and $D_{Au}=16$ nm. For the Au-PS PGN envisioned as core-shell particle, the quadrupolar frequency at 62.7 GHz is slightly red shifted mainly due to the larger PGN diameter (D=18.4 nm) and the PGN elasticity. Since bulk PS[9] has very close $c_{t,PS}$ (=1240m/s), the experimental value could be captured by $f(1,2)=0.94c_{t,eff}/D$ using about 10% larger value of the numerical prefactor. The modes at 59.7 GHz (Au in air) and 57.1 GHz (core-shell) using isotropic elasticity correspond to the torsional (1,2) spherical Lamb mode. For the isolated Au nanoparticle, the torsional mode (1,2) spherical Lamb mode is the eigenmode with the lowest non-zero frequency.

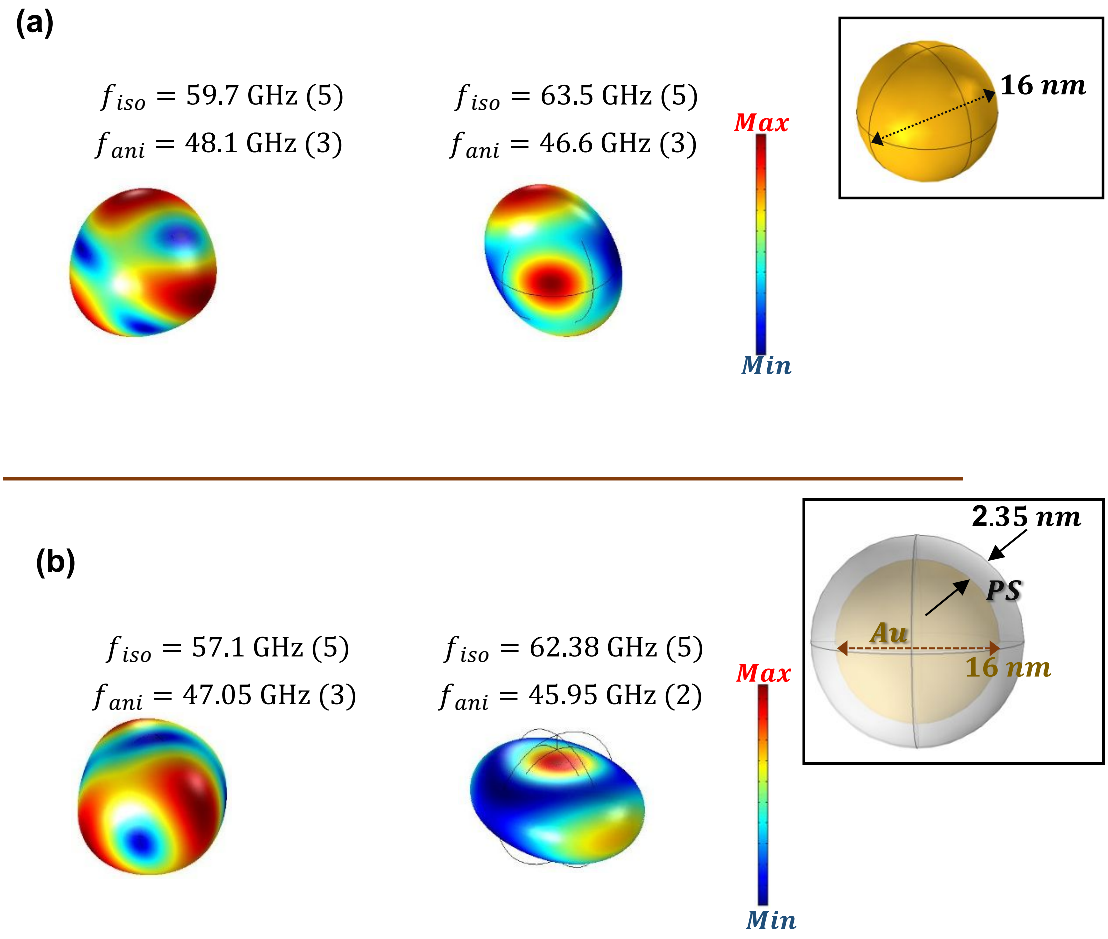

Figure S2: Finite element method-based simulation of bare and polystyrene (PS) covered Au nanoparticle in air. (a) Shape of the torsional and quadrupolar eigenmodes and their corresponding vibrational frequencies for bare Au nanoparticles in air. **(b)** Vibrational frequencies for Au-PS core-shell nanoparticles in air. The presence of the PS shell leads to a small red shift due to the similar transverse sound velocities of Au and PS. In both panels, the colormaps indicate the mean displacement. For each eigenmode, we provide the frequency assuming isotropic (f_{iso}) or anisotropic (f_{ani}) elasticity of Au. The integers in parentheses indicate the degeneracy of each mode. The geometry for each model is shown as an inset.

S3: Depolarized (VH) spectrum of Au-PS monolayer on Si substrate.

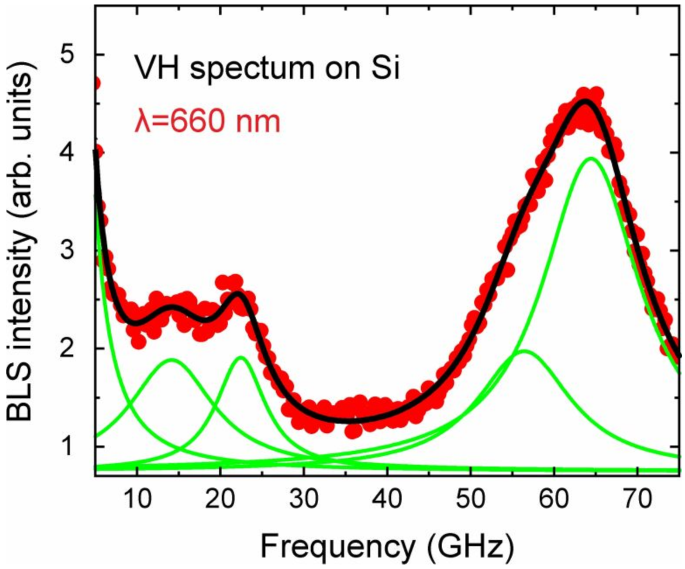

Figure S3: Depolarized BLS spectra (VH) of Au-PS nanoparticles on Silicon. The BLS spectrum of Au-PS monolayer on Si substrate recorded with VH polarization using laser light at 660 nm at 3 mW. The solid lines indicate the representation of the experimental spectrum (points) with the sum (black) of four Lorentzian peak profiles (solid green lines). The positions of the Lorentzian peaks are listed in **Table 1** of the main manuscript. The first two plasmon-enhanced low-frequency peaks appear at f_1=14.3 ± 0.3 GHz and f_2 =22.4 ± 0.2 GHz with intensity ratio, I_1/I_2 = 0.6. The broad asymmetric peak at ~60 GHz is represented by two Lorentzians at f_3 =56.4 ± 0.6 and f_4 =64.4 ± 0.2 GHz with intensity ratio, I_3/I_4 = 0.37. The intensity ratio of the two low-frequency peaks ($I_{1,2}$) to the two high-frequency peaks ($I_{3,4}$) is $I_{1,2}/I_{3,4}$=0.36.

S4. Brillouin light scattering measurements on SiO_2 glass.

The Brillouin Light Scattering (BLS) measurements of **Figure 2** have been repeated for the same Au-PS PGN monolayer on SiO_2 glass substrate, and the spectra are shown in **Figure S4**. The glass substrate has lower heat conductivity than Si, and thus we have lowered the incident laser power to ~2.5 mW to avoid any laser-induced structural changes. Additionally, we suppressed any laser-induced structural changes by measuring multiple spots with short exposure time and averaging the spectra. The angle of incidence was 45° and the wavelengths of light were 532 nm (**Fig. S4a,b**) and 660 nm (**Fig. S4c,d**). The experimental BLS spectra

(points) were represented with Lorentzian peak profiles (solid black lines) – unless stated otherwise.

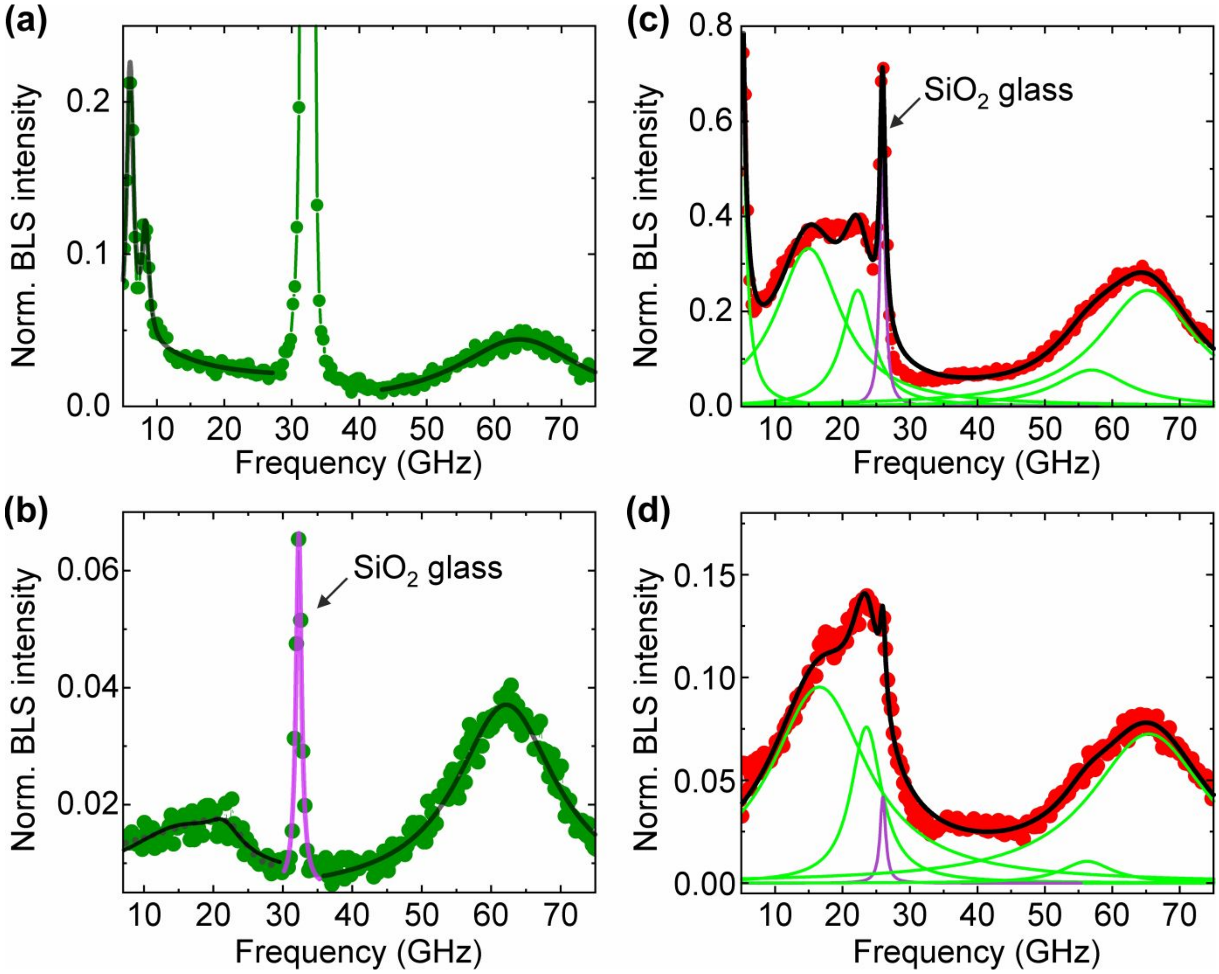

Figure S4: BLS spectra of polystyrene brushed Au nanoparticles (PGN) on glass substrate. BLS spectra recorded at λ = 532 nm at HH **(a)** and VH **(b)** polarization, and at λ = 660 nm at HH **(c)** and VH **(d)** polarization. The measurements were performed at an incident angle of 45° at ~ 2.5 mW laser power. The sharp peak attributed to SiO_2 denotes the frequency of the longitudinal phonon in the glass substrate probed at backscattering (see text for more details). In **(b)** the low-frequency components were represented either by an asymmetric double sigmoidal curve (grey dotted line) or a double Lorentzian (black solid line) in order to estimate the frequency and intensity of these modes.

The HH spectrum measured with 532 nm (**Fig. S4a**) displayed two narrow peaks at 6 and 8.3 GHz. These peaks appeared only at HH polarization and vanished at VH (**Fig. S4b**) were attributed to surface acoustic waves, SAWs. Taking into account the angle of incidence (45°) the frequencies 6 and 8.3 GHz correspond to SAWs with phase velocities 2260 m/s and 3120

m/s, respectively. These phase velocities are in excellent agreement with band structure measurements and calculations (see **Figure S5b** and main article). Other than a broad background, no other peaks were resolved with HH polarization up to 30 GHz at 532 nm. In the frequency range 30-35 GHz, the HH spectrum was dominated by the SiO_2 glass peak, while the quadrupolar-like peak appears at 63.8 GHz. The BLS spectra recorded at 532 nm light and VH polarization (**Fig. S4b**) displayed a broad feature at ~20 GHz and they had a significantly lower intensity of the glass peak (magenta solid line). The broad asymmetric feature at ~20 GHz could be represented with an asymmetric double sigmoidal function centered at ~19 GHz (dashed grey line) or a double Lorentzian at ~15 and 21 GHz. At 532 nm, the quadrupolar-like mode at 62 GHz displayed a single Lorentzian shape. The intensity ratio of the low frequency modes at ~20 GHz (I_{Lf}) to the high frequency peak at 63.8 GHz (I_{Hf}) was $I_{Lf} / I_{Hf} \approx 0.34$.

At 660 nm and HH polarization (**Fig. S4c**), we observed plasmon-enhanced BLS peaks located at f_l=15.0 ± 0.2 GHz and f_2=22.3 ± 0.1 GHz. The broad peak at ~60 GHz could be represented with two Lorentzian peak profiles at f_3=56.7 ± 0.4 GHz and f_4=64.6 ± 0.3 GHz with intensity ratio I_3/I_4~0.24. The intensity ratio of the low frequency modes (I_{Lf}) to the high frequency modes (I_{Hf}) has increased to $I_{Lf} / I_{Hf} \approx 0.9$ compared to $I_{Lf} / I_{Hf} \approx 0.34$ at 532nm.

The BLS spectrum of Au-PS monolayers with VH polarization (**Fig. S4d**) also displayed plasmon-enhanced low frequency peaks at 16.6 ± 0.5 GHz and 23.5 ± 0.1 compared to the double Lorentzians at 56 ± 1 GHz and 65.4 ± 0.9 GHz represented by the intensity $I_{Lf} / I_{Hf} \approx 1.3$. For the two Lorentzian components at ~60 GHz, the intensity ratio (I_3/I_4) is in the order of ~0.1 indicating domination of the quadrupolar mode at 65.4 GHz. Conclusively, the BLS signal at 660 nm for the low-frequency plasmon-enhanced modes in PGN films on glass recorded is approximately equal to the BLS signal of the modes around 60 GHz. In the case of PGN films on Si, the intensity ratio is I_{Lf}/I_{Hf}=0.45 for HH in **Fig. 2d** and 0.36 for VH polarization in **Fig. S3**), which is more than twice lower. These observations indicate that the optical properties of the substrate are also affecting the optomechanical coupling and the BLS signal of the nanoparticle vibrations.

Finally, all spectra in **Figure S4** contained a narrow peak attributed to the longitudinal acoustic waves in the SiO_2 substrate. Since the measurements were performed at two different laser wavelengths, the probed wave vectors and hence the frequency of the SiO_2 peak changes.

S5. SAWs in Polystyrene brushed Au nanoparticle on glass substrate

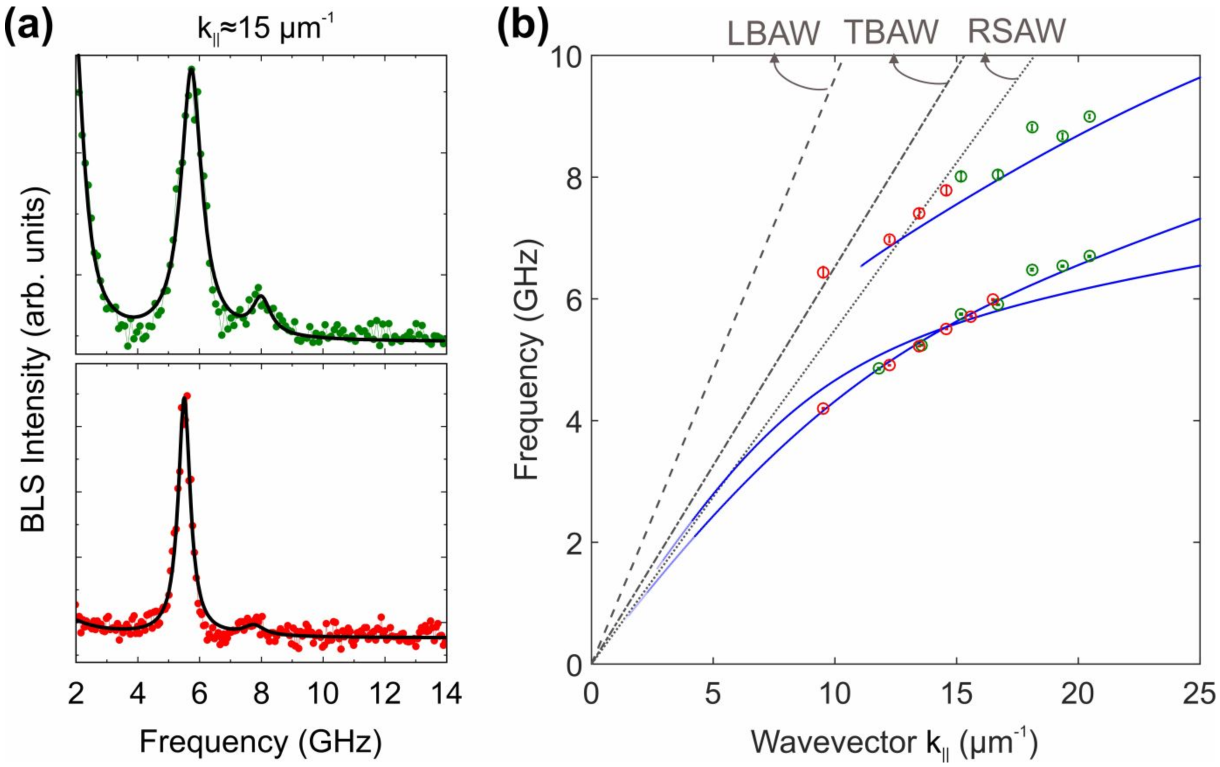

Figure S5: (a) Angle-resolved HH spectra at k~15 μm-1 for 532 nm (green, up) and 660 nm (red, down). **(b)** The theoretical dispersion of the SW obtained from Comsol simulations (lines) along with the experimental frequencies of the two SW modes (symbols). Theoretical lines calculated for longitudinal bulk acoustic wave (LBAW), transverse bulk acoustic wave (TBAW), and Rayleigh surface acoustic wave (RSAW) in SiO_2 are demonstrated by additional dashed lines.

S7. Elasticity of effective thin films

The good comparison between the FEM calculations and the measured dispersion relationships of true-SAWs can be used to gain a deeper understanding of the mechanical properties of PGNs. For our samples, the Au core diameter (D=16 nm) and the total particle diameter together with the PS chains ($D_T = 20.7$ nm) gives the volume fraction of Au: $\varphi_{\mathrm{Au}} = (D/D_T)^3 = 0.46$. The nanoparticle density is:

$$\rho_{\mathrm{NP}} = \varphi_{\mathrm{Au}}\,\rho_{\mathrm{Au}} + (1-\varphi_{\mathrm{Au}})\,\rho_{\mathrm{PS}} = 9.4\ \mathrm{g/cm^3}\,, \quad (1)$$

where $\rho_{\mathrm{Au}} = 19.3$ g/cm^3 and $\rho_{\mathrm{PS}} = 1.04$ g/cm^3 are the densities of Au and PS, respectively. The PGNs form closed thin films with a hexagonal arrangement of the crystalline cores. The process involves the stretching of the PS chains to fill the holes of the hexagonal lattice. Therefore, the final density of the superlattice (ρ_{SL}) should be:

$$\rho_{\mathrm{SL}} \cong 0.74\, \rho_{\mathrm{NP}} + (1 - 0.74) \cdot \rho_{\mathrm{m}}, \quad (2)$$

where the 0.74 stands for the filling factor of an FCC lattice and ρ_{m} for the density of the surrounding matrix. Assuming $\rho_m = \rho_{\mathrm{PS}}$ we get $\rho_{\mathrm{SL}} = 7.23$ g/cm^3. In the actual samples, the matrix surrounding the nanoparticles is made of stretched PS chains, which are expected to have a decreased density compared to the bulk polymer. Thus, the simple calculation with Relationship (2) is in excellent agreement with the value that was assumed in the FEM calculations (~7.02 g/m^3) in order to reproduce the experimental dispersion relationships. The value for the Poisson ratio that we assumed (0.37) is in between the values for Au (0.415) and PS[10] (0.32). Finally, we comment on the estimated value for the Young modulus.

Both PS and Au have similar transverse speed of sound: $c_t \cong 1200$ m/s. The shear modulus for Au and PS is $G_{\mathrm{Au}} = \rho_{\mathrm{Au}} c_t^2 = 27.8$ GPa and $G_{\mathrm{PS}} = \rho_{\mathrm{PS}} c_t^2 = 1.5$ GPa, respectively. Based on the inverse law of mixtures, the shear modulus of the polymer-grafted particle (G) obeys:

$$\frac{1}{G} = \frac{\varphi_{\mathrm{Au}}}{\rho_{\mathrm{Au}} c_t^2} + \frac{1 - \varphi_{\mathrm{Au}}}{\rho_{\mathrm{PS}} c_t^2} \,. \quad (3)$$

The last relationship gives $G = 2.65$ GPa and the corresponding value for the Young modulus is $E = 2G(1 + \nu) = 7.26$ GPa, which captures very well the value used to represent the recorded dispersion relationships (**Fig. 3b**).

S8. Bare Au nanoparticle on Si substrate

The plasmon-enhanced peaks at low frequencies (~15-25 GHz), which appear for both HH and VH polarization are attributed to plasmon-enhanced coupling modes between nanoparticles, whereas the narrow peaks at ~10 GHz or less that appear only for HH polarization are attributed to SAWs. The existence of these SAWs suggests that the monolayer of the polymer-grafted nanoparticles acts effectively as a thin-film at long phonon wavelengths. In **Figure S6**, we show the BLS spectra of a 1-Dodecanethiol-capped gold nanoparticles of the same diameter (D_{Au}=16 nm). Noticeably, in this case, the SAWs peaks vanish for both 532 nm (**Fig. S6a**) and 660 nm

(**Fig. S6b**) albeit at HH polarization. The most prominent peak in these BLS spectra (**Figs. S6a,b**) is a broad asymmetric peak centered around ~65 GHz. This spectral feature can be represented by two Lorentzian peaks with f_3=(55.1 ±0.4) GHz and f_4=(63.9±0.1) GHz and intensity ratio I_3 /I_4 =0.3 being experimentally the same with the ratio (0.29) for the PGN monolayer film in **Fig. 2d**.

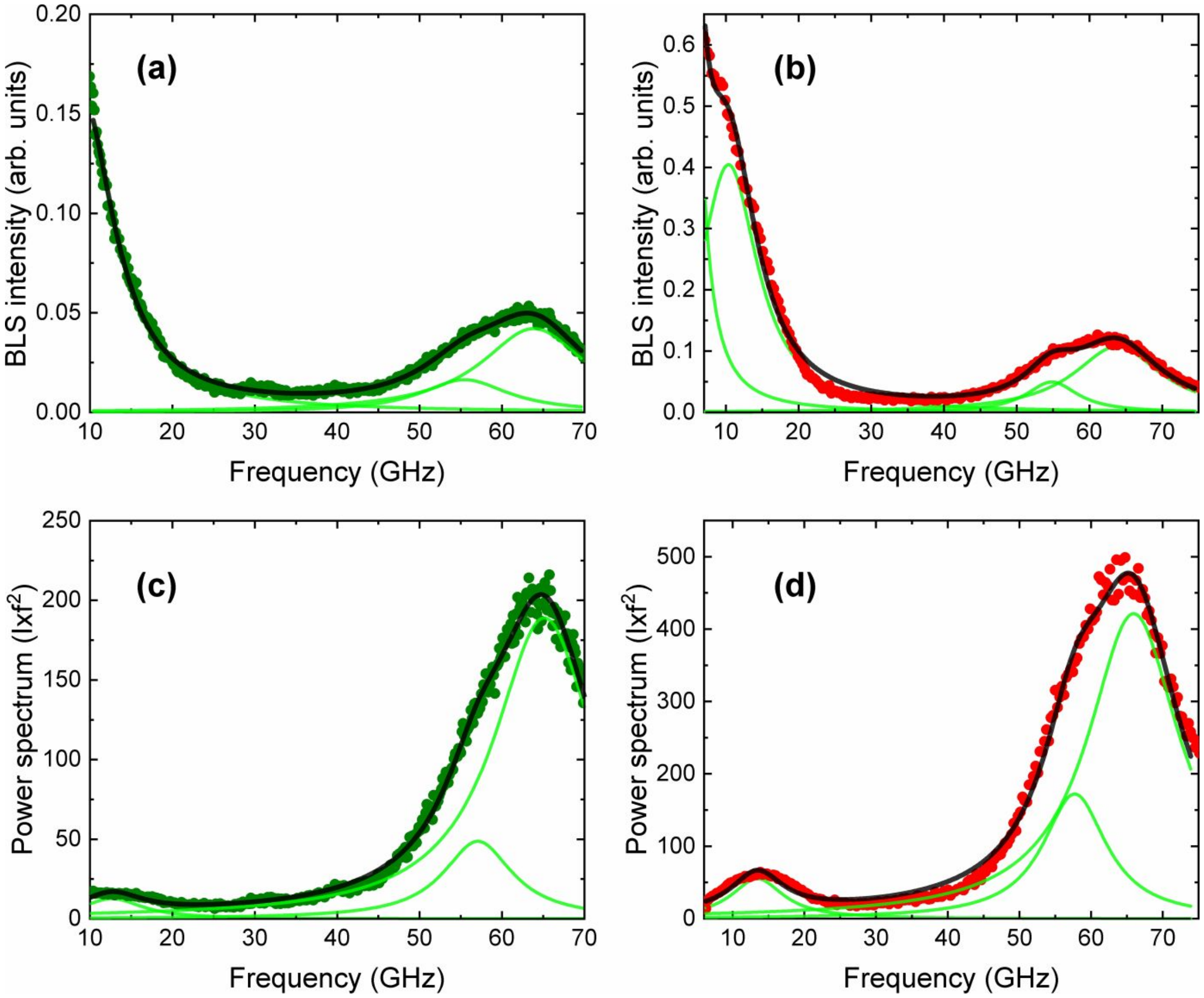

Figure S6: BLS measurements of bare (1-Dodecanethiol-capped) gold nanoparticles on Si substrate at HH polarization. (a) Experimental BLS spectrum (green points) recorded at 532 nm and its representation with the sum (black solid line) of Lorentzian peaks profiles (green solid lines). **(b)** Experimental BLS spectrum (red points) recorded at 660 nm and its representation with the sum (black solid line) of Lorentzian peaks profiles (green solid lines). The power spectra of **(a)** and **(b)** are shown in **(c)** and **(d)**, respectively. All spectra were recorded with 3 mW incident power, HH polarization, and 45° angle of incidence. For the powder-like samples shown here the nanoparticles are no longer forming a well-defined thin-film and the SAWs peaks disappear.

The BLS spectra of the bare Au nanoparticles recorded at 532nm (**Fig. S6a**) and 660nm (**Fig. S6b**) have a broad Rayleigh wing. Any peak profile hidden near the Rayleigh can be revealed with the BLS power spectrum ($I \cdot f^2$) shown in **Figures 6c** and **6d**, respectively at 532 nm and 660 nm. Notably, the broad background at <30 GHz forms a peak in the BLS power spectra, which is enhanced at 660 nm (**Fig. 6d**) compared to 532 nm (**Fig. 6c**). This broad peak in the power spectra is located at approximately 12.7 GHz at 532 nm and 13.4 GHz at 660 nm. The observations for the 1-Dodecanethiol-capped gold nanoparticles, which have a powder-like morphology, support the hypothesis that couplings between nanoparticles create the broad low-frequency peaks visible at VH. For bare Au and 660 nm, the intensity of the low frequency coupling mode is about twice the total intensity of the two Lorentzians around 60 GHz (**Fig. 6Sb**). For comparison, the intensity ratio of the two low-frequency coupling modes to the high-frequency modes of Au-PS nanoparticles on Si recorded at 660 nm is about 0.4 (0.45 for HH polarization in **Fig. 2b** and 0.36 for VH polarization in **Fig. S3**). Thus, the BLS activity of the low-frequency coupling modes increases as the interparticle gap decreases.

S9. Plasmon-enhanced coupling modes in Au-PS dimers

The simulations of **Figure S7** show how the mechanical and plasmonic coupling in dimers of Au-PS nanoparticles can create BLS active modes at ~10-25 GHz through mechanical and plasmonic coupling. Moreover, the mechanical coupling of the dimer can lift the degeneracy of the quadrupolar mode and lead to broadening of the 63 GHz-peak. The geometry of the model for the dimer is shown in **Figure S7a**. Two Au nanoparticles 4.7 nm apart are embedded in a PS matrix. The PS matrix has a spherical shape and it is enclosed in perfectly matched layers (PML) to absorb outgoing acoustic and electromagnetic waves. Due to the PS matrix there are multiple modes that are irrelevant to the dimer. To distinguish the eigenmodes of the dimer from the waves in the PS matrix, we define the localization rate as $L=U_{NP}/U_{total}$. The U_{NP} and U_{total} are the elastic energies in the nanoparticles and in the entire simulated volume, respectively. In this way, we obtain the spectrum of eigenmodes shown in **Figure S7b**. The first three most prominent modes up to 17 GHz are rattling modes. Moreover, we observe two coupled torsional modes around 20 GHz. Noticeably, for the bare Au nanoparticle there are no elastic excitation between 5-30 GHz, meaning that the rattling and torsional modes appear because of the PS-mediated mechanical coupling. Interestingly, the mechanical coupling is also affecting the quadrupolar mode as shown in **Figure S7c**. Each quadrupolar mode for the bare Au nanoparticle has degeneracy 5. In the dimer, the quadrupolar modes can couple with different orientations and phases giving rise to a forest of peaks in the 60-70 GHz range.

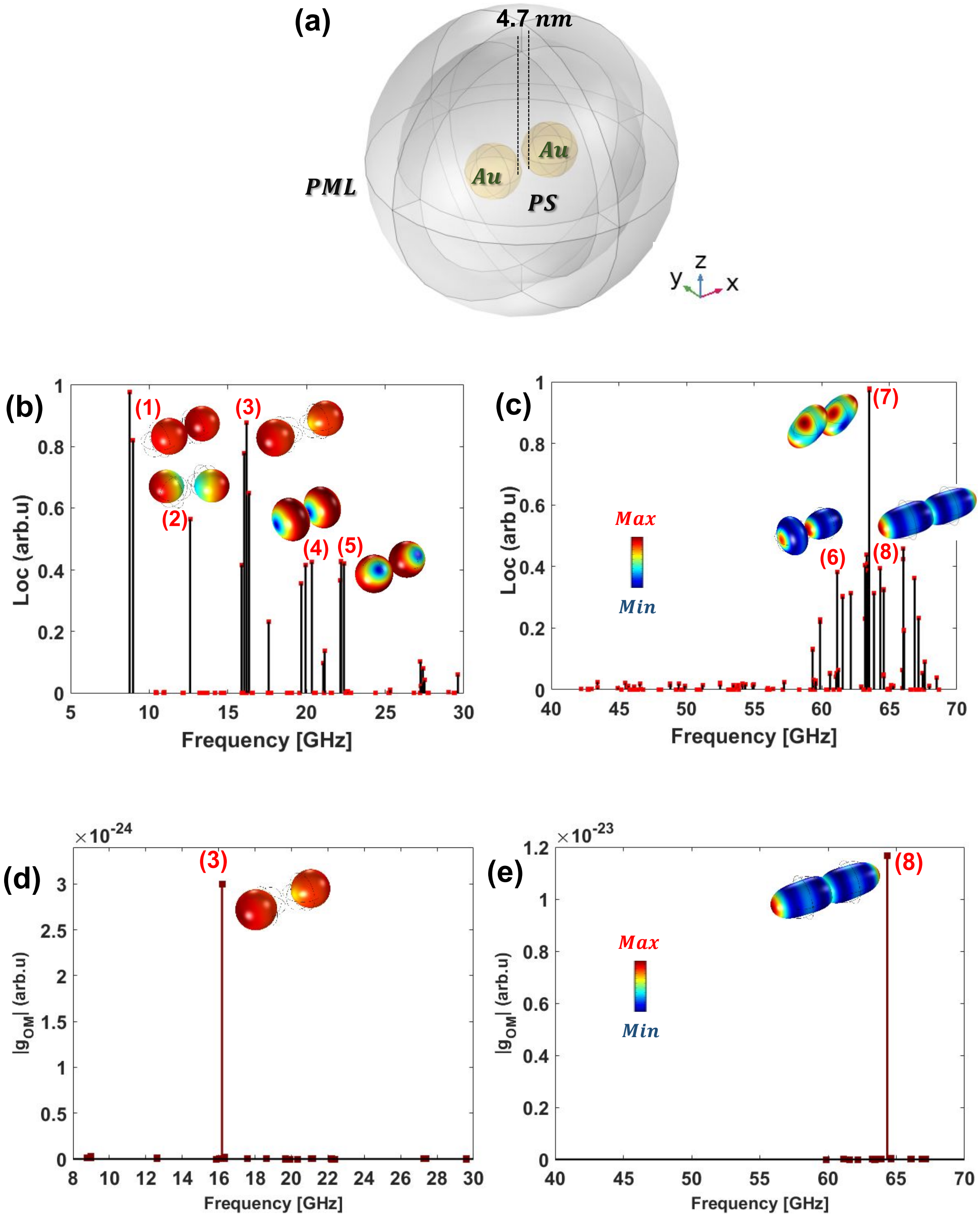

Figure S7: Finite element method (FEM) calculation of vibrational eigenmodes for a 4.7 nm-separated gold dimer in a PS matrix and their optomechanical (OM) coupling with a 660 nm polarized along the dimer axis. **(a)** Geometry model used for computing OM coupling and eigenmodes. **(b, c)** Localization rate of eigenmodes as a function of eigenfrequency in the low and high-frequency regions, respectively, with insets showing mode shapes (based on displacement field norm) for the most localized modes. **(d, e)** Corresponding OM coupling

coefficients versus eigenfrequency for the low and high-frequency regions. Notably, the out-of-phase rattling mode at ≈16 GHz (low frequency) and in-phase quadrupolar modes at ≈64 GHz exhibit the strongest OM coupling.

To calculate the optomechanical (OM) coupling accurately, it is crucial to consider electromagnetic (EM) quasi-eigenmodes in plasmonic nanoparticles, which are dispersive and dissipative. Following Primo et al.[11], these modes preserve the orthogonality needed to derive OM coupling via perturbation theory. Since the BLS experiments use selected wavelengths of light, rather than exciting selected EM eigenmodes, we adopt the approach of Bai et al.[12] and treat the scattered electric field distribution as a quasi-eigenmode of the system. This is valid because the wavelengths used in this work (532 and 660 nm) lie near resonances of plasmonic oscillations in individual nanoparticles (~527 nm) and bright coupled modes (~610 nm), allowing the scattered field to be well approximated by a dominant quasi-eigenmode for computing optomechanical coupling.

To identify which vibrational modes are BLS active, we performed finite element method calculations of acoustic phonons, plasmons, and optomechanical coupling[13]. In this way, we obtained the optomechanical coupling strength $|g_{OM}|$ for each eigenmode. As described in the main article, the dominant source of $|g_{OM}|$ is the moving interface (MI) effect. An additional contribution comes from the photoelastic effect (PE), which occurs within the volume of the material. The PE coupling is given by an integral of the form:

$$g_{PE} \propto \iiint E_i^* \, \delta\tilde{\varepsilon} \, E_s \, dV \,, \quad (4)$$

where, E_i^* is the complex conjugate of the incident electric field, E_s is the scattered electric field, and $\delta\tilde{\varepsilon}$ is the amount by which the permittivity of gold is modified due to the acoustic vibration[14]. The PE contribution is weak for plasmonic nanostructures, since $E_i^* \cong 0$ within the volume, V, of the metal. Furthermore, any weak signal associated with PE is expected to be significantly broadened due to optical losses in gold.

The calculated $|g_{OM}|$-spectrum for the PGN monolayer on SiO_2 glass is shown in **Figure S7d** for the low-frequency coupling modes, and in **Figure S7e** for the quadrupolar-like modes. In the case of the dimer, the modes with the strongest OM coupling modify strongly the interparticle gap, i.e., a rattling mode at 16 GHz and a coupled quadrupolar mode at ~65 GHz.

In the case of the PGN monolayers the results are more complicated due to the existence of 6 nearest neighbours and the translational symmetry of the system.

S9. Phononic band structure and optomechanical coupling of PGN monolayers on glass.

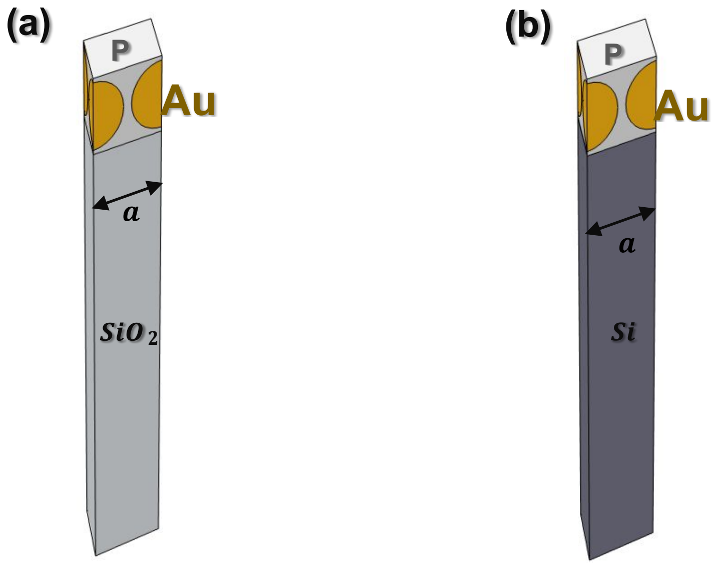

Figure S8: Unit cells for the calculations of dispersion curves. The primitive unit cell of the monolayer was placed on top of a thick slab of SiO_2 glass **(a)** or crystalline Si **(b)**.

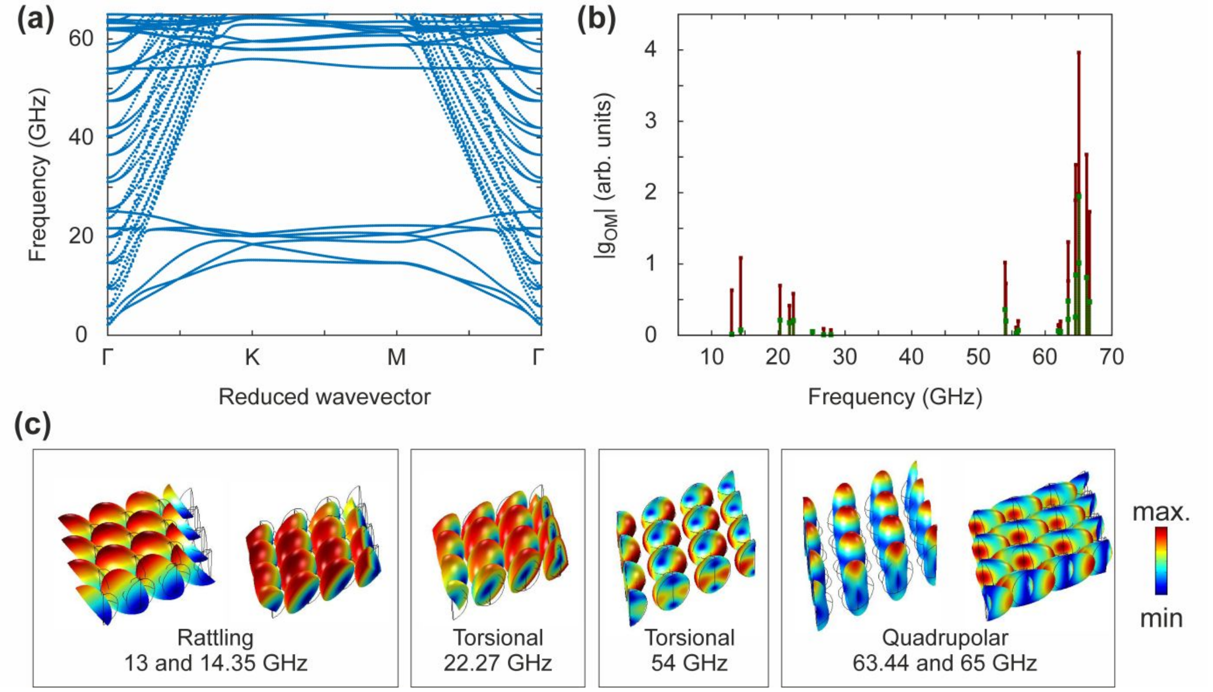

Figure S8: Band structure and optomechanical coupling of coupled nanoparticle modes on SiO_2 glass. (a) The band structure of coupled PGN modes across the high-symmetry k-

points of the hexagonal lattice. **(b)** The optomechanical coupling for 532 nm light (green stems) and 660 nm (red stems). The parallel wavevector is $k_{||}$ = 16.7 μm^{-1} for λ = 532 nm, and $k_{||}$ = 13.5 μm^{-1} for λ = 660 nm. **(c)** The displacement fields of the BLS active modes. The parallel wavevector is set to $k_{||}$ = 13.5 μm^{-1} in the Γ-to-M direction.

References

(1) Zhang, J.; Santos, P. J.; Gabrys, P. A.; Lee, S.; Liu, C.; Macfarlane, R. J. Self-Assembling Nanocomposite Tectons. *J Am Chem Soc* **2016**, *138* (50). https://doi.org/10.1021/jacs.6b11052.

(2) Santos, P. J.; Gabrys, P. A.; Zornberg, L. Z.; Lee, M. S.; Macfarlane, R. J. Macroscopic Materials Assembled from Nanoparticle Superlattices. *Nature* **2021**, *591* (7851). https://doi.org/10.1038/s41586-021-03355-z.

(3) Santos, P. J.; Macfarlane, R. J. Reinforcing Supramolecular Bonding with Magnetic Dipole Interactions to Assemble Dynamic Nanoparticle Superlattices. *J Am Chem Soc* **2020**, *142* (3). https://doi.org/10.1021/jacs.9b11476.

(4) Turkevich, J.; Stevenson, P. C.; Hillier, J. A Study of the Nucleation and Growth Processes in the Synthesis of Colloidal Gold. *Discussions of the Faraday Society*. 1951. https://doi.org/10.1039/DF9511100055.

(5) Schulz, F.; Homolka, T.; Bastús, N. G.; Puntes, V.; Weller, H.; Vossmeyer, T. Little Adjustments Significantly Improve the Turkevich Synthesis of Gold Nanoparticles. *Langmuir* **2014**, *30* (35). https://doi.org/10.1021/la503209b.

(6) Bastús, N. G.; Comenge, J.; Puntes, V. Kinetically Controlled Seeded Growth Synthesis of Citrate-Stabilized Gold Nanoparticles of up to 200 Nm: Size Focusing versus Ostwald Ripening. *Langmuir* **2011**, *27* (17). https://doi.org/10.1021/la201938u.

(7) Park, Y. K.; Yoo, S. H.; Park, S. Assembly of Highly Ordered Nanoparticle Monolayers at a Water/ Hexane Interface. *Langmuir* **2007**, *23* (21). https://doi.org/10.1021/la701445a.

(8) Vasileiadis, T.; Noual, A.; Wang, Y.; Graczykowski, B.; Djafari-Rouhani, B.; Yang, S.; Fytas, G. Optomechanical Hot-Spots in Metallic Nanorod-Polymer Nanocomposites. *ACS Nano* **2022**, *16* (12). https://doi.org/10.1021/acsnano.2c06673.

(9) Cheng, W.; Sainidou, R.; Burgardt, P.; Stefanou, N.; Kiyanova, A.; Efremov, M.; Fytas, G.; Nealey, P. F. Elastic Properties and Glass Transition of Supported Polymer Thin Films. *Macromolecules* **2007**, *40* (20). https://doi.org/10.1021/ma071227i.

(10) Schneider, D.; Beltramo, P. J.; Mattarelli, M.; Pfleiderer, P.; Vermant, J.; Crespy, D.; Montagna, M.; Furst, E. M.; Fytas, G. Elongated Polystyrene Spheres as Resonant Building Blocks in Anisotropic Colloidal Crystals. *Soft Matter* **2013**, *9* (38). https://doi.org/10.1039/c3sm50959a.

(11) Primo, A. G.; Carvalho, N. C.; Kersul, C. M.; Frateschi, N. C.; Wiederhecker, G. S.; Alegre, T. P. M. Quasinormal-Mode Perturbation Theory for Dissipative and Dispersive Optomechanics. *Phys Rev Lett* **2020**, *125* (23). https://doi.org/10.1103/PhysRevLett.125.233601.

(12) Bai, Q.; Perrin, M.; Sauvan, C.; Hugonin, J.-P.; Lalanne, P. Efficient and Intuitive Method for the Analysis of Light Scattering by a Resonant Nanostructure. *Opt Express* **2013**, *21* (22). https://doi.org/10.1364/oe.21.027371.

(13) Noual, A.; Akiki, R.; Lévêque, G.; Pennec, Y.; Djafari-Rouhani, B. Enhanced Phonon-Plasmon Interaction in Film-Coupled Dimer Nanoridges Mediated by Surface Acoustic Waves. *Opt Express* **2021**, *29* (26). https://doi.org/10.1364/oe.444430.

(14) Ahmed, A.; Pelton, M.; Guest, J. R. Understanding How Acoustic Vibrations Modulate the Optical Response of Plasmonic Metal Nanoparticles. *ACS Nano* **2017**, *11* (9). https://doi.org/10.1021/acsnano.7b04789.
